# The safety of artemisinins during pregnancy: a pressing question

**DOI:** 10.1186/1475-2875-6-15

**Published:** 2007-02-14

**Authors:** Stephanie Dellicour, Susan Hall, Daniel Chandramohan, Brian Greenwood

**Affiliations:** 1 Department of Infectious and Tropical Diseases, London School of Hygiene & Tropical Medicine, 50, Bedford Square, London, WC1B 3DP, UK; 2 Worldwide Epidemiology, GlaxoSmithKline, Research Triangle Park, NC 27709-3398, USA; 3 Department of Infectious and Tropical Diseases, London School of Hygiene &Tropical Medicine, Keppel Street, London, WC1E 7HT, UK; 4 Department of Infectious and Tropical Diseases, London School of Hygiene &Tropical Medicine, 50, Bedford Square London, WC1B 3DP, UK

## Abstract

**Background:**

An increasing number of countries in sub-Saharan Africa are changing to artemisinins combination therapy (ACT) as first or second line treatment for malaria. There is an urgent need to assess the safety of these drugs in pregnant women who may be inadvertently exposed to or actively treated with ACTs.

**Objectives:**

To examine existing published evidence on the relationship between artemisinin compounds and adverse pregnancy outcomes and consider the published evidence with regard to the safety of these compounds when administered during pregnancy.

**Methods:**

Studies on ACT use in pregnancy were identified via searches of MEDLINE, EMBASE, Cochrane and Current Contents databases. Data on study characteristics, maternal adverse events, pregnancy outcomes and infant follow up were extracted.

**Results:**

Fourteen relevant studies (nine descriptive/case reports and five controlled trials) were identified. Numbers of participants in these studies ranged from six to 461. Overall there were reports on 945 women exposed to an artemisinin during pregnancy, 123 in the 1st trimester and 822 in 2nd or 3rd trimesters. The primary end points for these studies were drug efficacy and parasite clearance. Secondary endpoints were birth outcomes including low birth weight, pre-term birth, pregnancy loss, congenital anomalies and developmental milestones. While none of the studies found evidence for an association between the use of artemisinin compounds and increased risk of adverse pregnancy outcomes, none were of sufficient size to detect small differences in event rates that could be of public health importance. Heterogeneity between studies in the artemisinin and comparator drugs used, and in definitions of adverse pregnancy outcomes, limited any pooled analysis.

**Conclusion:**

The limited data available suggest that artemisinins are effective and unlikely to be cause of foetal loss or abnormalities, when used in late pregnancy. However, none of these studies had adequate power to rule out rare serious adverse events, even in 2^nd ^and 3^rd ^trimesters and there is not enough evidence to effectively assess the risk-benefit profile of artemisinin compounds for pregnant women particularly for 1^st ^trimester exposure. Methodologically rigorous, larger studies and post-marketing pharmacovigilance are urgently required.

## Background

Every year over 50 million pregnancies occur in areas where malaria is endemic, mostly in sub-Saharan Africa. Malaria can have serious consequences for the mother and her baby. In regions where malaria transmission is unstable, pregnant women are at high risk of developing severe malaria, spontaneous abortion, stillbirth or premature delivery[1]. In high transmission regions, infected pregnant women are often asymptomatic but parasitaemia can cause maternal anaemia and low birth weight (LBW), a leading cause of infant morbidity and mortality. About 8–14% of LBWs and 3–8% of infant mortality in sub-Saharan Africa are attributable to pregnancy-associated malaria (PAM)[2].

The WHO recommends a three-pronged approach to control of malaria during pregnancy which includes effective case management, intermittent preventive treatment (IPT) and insecticide-treated nets (ITN). Until recently, WHO recommendations for case management during any trimester of pregnancy were chloroquine (CQ), or sulphadoxine-pyrimethamine (SP) in CQ-resistant areas and, alternatively, quinine in areas where neither were effective. There is limited information on the safety profile of most antimalarials used for treatment or prevention of malaria in pregnancy. In a period of increasing parasite resistance to inexpensive, conventional antimalarials such as chloroquine, SP and quinine, there is a pressing need to identify drugs which have the most favourable harm-benefit balance for these vulnerable patients [3,4]. Artemisinins are a potentially valuable alternative as they are highly effective, act rapidly and are well-tolerated. In addition, they have the potential to reduce the transmission of malaria and to slow development of resistance[5]. In 2002, after a detailed review of published and unpublished data, a WHO expert committee concluded that artemisinins could be used during the second or third trimesters if no suitable alternative was available[6] However, treatment in the first trimester was not recommended unless the life of the woman was at risk because of concerns raised by animal experiments which suggested that artemisinins might be teratogenic and cause foetal resorption. Further studies have confirmed the embryotoxic effects of artemisinin and its derivatives in animals, including primates, with risk being confined to a defined period of gestation [7,8]. However it remains unknown how these findings translate to man. Because of these safety 'signals' from animal models, there is an urgent need to establish the safety profile of this class of drugs in pregnancy. By June 2006, 37 countries in Africa had adopted ACTs as the first or second line treatment policy [9] and consequently pregnant women are likely to be increasingly exposed to ACTs; many women will be exposed in the first trimester before they are aware of their pregnancy. This review examines all published and ongoing studies reporting exposure to artemisinins during pregnancy and discusses their safety during pregnancy.

## Methods

Electronic searches were made using MEDLINE/PubMed, EMBASE (1980 – July 2006) databases and through the Cochrane Central Register of Controlled Trials (CENTRAL). Reference lists from identified articles and conference abstracts, including those of the Multilateral Initiative on Malaria (MIM) conference, 2005, and the American Society of Tropical Medicine and Hygiene (ASTMH) annual meeting, 2005, were reviewed. The National Institute of Health's list of clinical trials was searched.

Studies on exposure of pregnant women with *Plasmodium falciparum *malaria to any artemisinin derivative in, irrespective of whether the participants were symptomatic, have been included in this review. The following data were extracted from reports: setting, year, study design, sample size, characteristics of study participants, drug regimen, and outcomes of interest. Outcomes of interests were: (1) maternal serious adverse events (fatal, life-threatening or requiring hospitalization); (2) maternal non-serious adverse events (3) adverse pregnancy outcomes (miscarriage, stillbirth, preterm delivery, low birth weight, neonatal death, congenital abnormality, developmental delay).

The internal and external validity of each study was assessed based on reporting comprehensiveness, the representativness of the study population, how bias and confounding factors were accounted for, and the power of the study.

### Description of studies

Sixty-nine studies of artemisinin-related drugs were identified. However, 55 reports were excluded because they were review papers, animal studies, or because study participants were not pregnant women. Among the fourteen studies that had reported maternal and foetal outcomes of treatment with an artemisinin derivative in pregnancy, five were randomized trials (RCT) and nine were descriptive non-randomized studies. Overall these represent 1,121 women who had been exposed to an artemisinin compound during pregnancy (242 in RCTs and 879 in observational studies). Taking into account inclusion of women in more than one of the studies reported from the Thai-Burmese border, reports were found of 945 pregnancies exposed to an artemisinin compound (123 in the 1st trimester and 822 in 2nd or 3rd trimesters). The design, characteristics of the study population, type of intervention and outcomes of each study included in the review are shown in Table 1.

**Table 1:** Description of the studies included in the review on the use of artemisinin in pregnancy.

**Study site, year Reference**	**Study Population**	**Antimalarial treatment**	**Primary Outcomes**
Randomized trials*			

Nigeria, 1994–1997 [14]	Pregnant women infected with *P. falciparum *malaria after treatment failure^§^	1) Artemether IM + mefloquine n = 22 2) Artemether IM n = 23 *Artemether overall dosage: 3.2–9.6 mg/kg*	Parasitaemia at day 28; AEs Foetal outcomes; infant neurodevelopment
Thailand, 1995–1998 [10]	Pregnant women infected with *P. falciparum *malaria^§^	1)Quinine n = 29 2)Artesunate+ mefloquine n = 28 *Artesunate overall dose: 12 mg/kg*	Time to parasite & fever clearance; anaemia; AEs; neurological examination Perinatal assessment; birth outcomes; infant physical and neurological assessment
TBB, 1995–1997 [12]	Pregnant women with uncomplicated *P. falciparum *malaria	1)Quinine n = 42 2)Artesunate+mefloquine n = 66 *Artesunate overall dose: 12 mg/kg*	Parasite clearance; anaemia; AEs; neurological deficit Birth outcomes; infant developmental milestones
TBB, 1997–2000 [13]	Pregnant women with uncomplicated *P. falciparum *malaria	1)Quinine+clindamycin n = 65 2) Artesunate n = 64 *Artesunate overall dose: 12 mg/kg*	Treatment failure at day 42 or before delivery; anaemia; AEs Birth outcomes; infant developmental milestones
TBB, 2001–2003 [11]	Pregnant women with uncomplicated *P. falciparum *or mixed malaria	1)Quinine n = 42 2) Artesunate atovaquone-proguanil n = 39 *Artesunate overall dose: 12 mg/kg*	Fever, parasite clearance; treatment failure at day 63; anaemia; AEs Birth outcomes; infant developmental milestones

***Case series***			

China, 1976–1980 [15]	Pregnant women infected with *P. falciparum *or *P vivax *malaria^§^ Trimesters (n): 3^rd ^(1), 2^nd ^(5)	Artemisinin IM or Artemether *Overall artemisinins dose: 500–900 mg*	Therapeutic effects and adverse reactions for mother and child
TBB, 1992–1996 [17]	Pregnant women with uncomplicated multi-drug resistant *P. falciparum *or mixed malarial infection Trimesters (n): 3^rd ^& 2^nd ^(74), 1^st ^(16)	Artesunate alone or with mefloquine; or Artemether+mefloquine *Overall artemisinins dose: 12–16 mg/kg*	Treatment failure at day 42; anaemia; ADR; neurological deficit Birth outcome; infant neurological development
TBB, 1991–1996 [18]	Pregnant women with uncomplicated multi-drug resistant *P. falciparum *malarial infection after treatment failure Trimesters (n): 3^rd ^& 2^nd ^(74), 1^st ^(16)	Artesunate alone or with mefloquine; or Artemether+mefloquine *Overall artemisinins dose: 12–16 mg/kg*	Treatment failure at day 42; anaemia; ADR; neurological deficit Birth outcome; infant neurological development
TBB, 1992–2000 [16]	Pregnant women with uncomplicated multi-drug resistant *P. falciparum *or mixed malarial infection Trimesters (n): 3^rd ^(211), 2^nd ^(201), 1^st ^(42)	Artesunate alone or with mefloquine; clindamycin atovaquone-proguanil;or coartemether, artemether IM *Overall artemisinins dose: 12–16 mg/kg*	Treatment failure at day 42; anaemia; ADR; neurological deficit Birth outcome; infant developmental milestones
TBB 1999–2001 [19]	Pregnant women with multi-drug resistant *P. falciparum *or mixed malarial infection Trimesters (n): 3^rd ^& 2^nd ^(24), 1^st ^(3)	Artesunate+atovaquone-proguanil *Artesunate overall dose: 12–14 mg/kg*	Treatment failure at day 42; parasite clearance; AEs Birth outcomes
TBB 2000–2001 [20]	Pharmacokinetic study: pregnant women with multi-drug resistant *P. falciparum *or mixed malarial infection Trimesters (n): 3^rd ^(13) & 2^nd ^(11)	Artesunate+atovaquone-proguanil *Artesunate overall dose: 12 mg/kg*	Pharmacokinetic parameters; AEs (including ECG); parasite clearance Birth outcomes
Gambia, 1999 [41]	Pregnant women participating in mass prevention campaign Trimesters (n): 1^st ^(77)	Artesunate + SP *Artesunate overall dose: 200 mg*	MDA 1° aim: malaria transmission Pregnancy outcomes
Sudan, 1997–2001 [22]	Pregnant women infected with *P. falciparum *malaria after treatment failure^§^ Trimesters (n): 3^rd ^(15), 2^nd ^(12), 1^st ^(1)	Artemether IM *Artemether overall dose: 480 mg*	Treatment failure at day 28; anaemia; neurological deficit Birth outcomes
Sudan, 2004–2005 [23]	Pregnant women infected with uncomplicated *P. falciparum *malaria^§^ Trimesters (n): 3^rd ^(22), 2^nd ^(10)	Artesunate + SP *Artesunate overall dose: 200 mg*	Treatment failure at day 28; anaemia Birth outcomes

* 2nd and 3rd trimesters only

§100% symptomatic/febrile

Abbreviation: ADR: adverse drug reaction; AE: adverse event; ECG: electrocardiograph; IM: intramuscular injection; NR: not reported; Q: quinine; A: artemisinin derivative; A+M: artesunate+mefloquine; TBB: Thai-Burmese border

### Randomized controlled trials

Five RCTs of artemisinin in pregnant patients have been reported [10-14]. A randomized trial in Nigeria studied 45 pregnant women treated with artemether alone or in combination with mefloquine, but provided only limited information regarding the safety of artemether because of inconsistent infant follow up[14]. In Thailand, an effectiveness and safety trial compared artesunate plus mefloquine with quinine in 57 pregnant women. This study excluded women who had taken antimalarials within the previous 28 days and those who had over 4% parasitized red cells. The study population comprised predominantly milder cases. Specific neurological evaluation of mothers was performed weekly using established tests and infants were assessed for physical and neurological development at 12 or 24 months [10]. Three of the comparative trials were conducted in antenatal clinics (ANCs) on the Thai Burmese Border. They compared different drug regimens of artemisinin or its derivatives to quinine in second or third trimester pregnancies. In 2000, a report of 108 pregnant women randomized to receive quinine or artesunate plus mefloquine was published [12]. This was followed in 2001 by a report of a randomized trial of artesunate versus quinine plus clindamycin in 129 pregnant women [13]. More recently, the results of a trial of 81 pregnant women randomized to receive quinine or artesunate-atovaquone-proguanil has been published [11]. Women who presented with a first malaria episode were recruited excluding those with complicated or severe malaria. Detailed information on outcome was available. A high level of efficacy of the artemisinin derivatives was reported in each study and the drugs were well tolerated.

### Descriptive studies

The first descriptive report of the use of artemisinin derivatives during pregnancy came from China. Wang *et al *reported the outcomes of six pregnancies exposed to either artemisinin or artemether. Children of these women were examined 5 to 10 years later for congenital malformation, physical and neurodevelopmental evaluation. No adverse events were reported for the mother or the infants [15].

McGready *et al *reported the outcome of case-series pregnancies exposed to artemisinins on the Thai-Burmese border in three succeeding publications [16-18]. The latest publication of 2001 encompasses all cases seen between 1992–2000 (461 women with 539 artemisinin-based treatments) including women described in previous publications [12,13]. Overall, there was 11% loss to follow up. A subgroup of 44 women was exposed inadvertently during their first trimester. In addition, two publications report on the treatment of 27 pregnant patients with atovaquone-proguanil plus artesunate[19,20]. The second publication [20], focuses on the pharmacokinetics properties of the drugs in 24 of these women. This group of studies provides the largest and most detailed record on the use of artemisinins in pregnancy.

During a mass drug administration campaign in the Gambia, 287 pregnant women were accidentally exposed to treatment with artesunate plus SP, including 77 who were exposed during the first trimester of pregnancy [21]. Both active and passive surveillance were used to maximize ascertainment of pregnancy outcomes. Reliable case ascertainment and outcome measurement was available for births notified within seven days of delivery, as these newborns were thoroughly examined by a paediatrician.

In Sudan, Adam *et al *have reported on two studies [22,23]. The first consisted of the follow-up of 28 symptomatic pregnant women who received intramuscular artemether after previous treatment failure with CQ or quinine. Women were assessed for neurological defects. There was one first trimester exposure which resulted in a normal newborn. The second study described 32 symptomatic pregnant women treated with artesunate plus SP. In both studies, women and their infants were followed up systematically (at birth and one year of age).

Ashley *et al *reported accidental exposure to artemisinin compounds during pregnancy in three clinical trials for the treatment of uncomplicated *P. falciparum *malaria [24]: they documented the inadvertent exposures to dihydroartemisinin-piperaquine of one woman at 11 weeks gestation and another at 18 weeks gestation [25,26]. In Uganda, four pregnant women were accidentally exposed in their 1st trimester to artemether-lumefantrine during a trial [27]. All these women delivered normal babies.

## Results

Maternal adverse events and pregnancy outcomes in the artemisinin group and comparison groups (where applicable) are summarized in Tables 2 and 3 respectively.

**Table 2:** Safety outcomes: maternal adverse events and pregnancy outcomes in women exposed to artemisinin compounds. Randomized trials

**Study site, year** **Ref**	**Antimalarial treatment**	**N (% follow up)**	**Outcomes of interests**	
			
			**Maternal Safety^¥^**	**Pregnancy outcomes**
Nigeria, 1994–1997 [14]	1) Artemether IM + mefloquine n = 22 2) Artemether IM n = 23	45 (100%)	Minimal, only A+M: abdominal discomfort (9%) and dizziness (9%)	Neonatal jaundice (n = 2 A & n = 1 A+M) 6 (13%) followed up to 1 year
Thailand, 1995–1998 [10]	1) Quinine n = 29 2) Artesunate+ mefloquine n = 28	60 (95%)	Neurological exam: all normal. Nausea (16%), vomiting (12%), vertigo (12%), tinnitus (18%), and hypoglycaemia (3%) more frequent in Q (p < 0.05). Other no difference: Palpitation (6%), blurring vision (11%).	Neonatal jaundice (n = 5 Q & n = 1 A+M) 46 (81%) followed up to 1 year
TBB, 1995–1997 [12]	1) Quinine n = 42 2) Artesunate+ mefloquine n = 66	108 (85%)	Neurological exam 1 maternal death* Tinnitus (15%) and dizziness (42%) more frequent in Q (p < 0.05). Headache (21%), nausea (45%), abdominal pain (28%), vertigo (12%), muscle/joint pain (32%), and anorexia (35%) no difference with Q (p > 0.05).	Abortions (n = 2 A+M) 46 (49%) followed up to 1 year Neonatal deaths (n = 2 A+M & n = 1 Q)
TBB, 1997–2000 [13]	1) Quinine)+ clindamycin n = 65 2) Artesunate n = 64	129 (91%)	Tinnitus (9%) more frequent in Q+C (p < 0.05). Headache (30%), dizziness (41%) nausea (25%), vomiting (8%), abdominal pain (18%), rash (9%), contractions (35%), muscle/joint pain (12%), and anorexia (42%) no difference with Q+C (p > 0.05).	Stillbirths (n = 1 A & n = 1 Q+C) Congenital abnormality: midline epidermoid cyst (n = 1 Q+C) Neonatal deaths (n = 1 A & n = 2 Q+C) 72 (62%) followed up to 1 year
TBB, 2001–2003 [11]	1)Quinine n = 42 2) Artesunate atovaquone-proguanil (AAP) n = 39	81 (91%)	1 maternal death** Tinnitus (24%) more frequent in Q (p < 0.05).	Stillbirth (n = 1 not specified maternal death) Congenital abnormalities: polythelia (n = 1 AAP); cleft lip & palate (n = 1 AAP); aural atresia (n = 1 Q) Neonatal deaths (n = 1 A & n = 2 Q+C) 59 (78%) followed up to 1 year Developmentally delayed (n = 1 AAP)
**TOTAL**	**No 1^st ^trimester, *P. falciparum *or mixed malaria 17% to 100% symptomatic 242 artemisinins exposures**	**423 (94%)**	**2 maternal deaths** **Neurological exam in 2 studies**	**Stillbirths (n = 1 A; n = 1 Q+C & n = 1 unknown)** **Congenital abnormality: (n = 2 A & n = 2 Q)** **Neonatal deaths (n = 4 A+M & n = 5 Q)** **229 (59%) infant followed up to 1 year**

^¥ ^Prevalence of possible ADRs are only reported for the artemisinin treatment groups

* cause unrelated to malaria (treatment group NR)

** caused by a ruptured liver abscess (treatment group NR)

Abbreviation: ADR: adverse drug reaction; AE: adverse event; IM: intramuscular injection; NR: not reported; Q: quinine; A: artemisinin derivative; A+M: artesunate+mefloquine; TBB: Thai-Burmese border

**Table 3:** Safety outcomes: maternal adverse events and pregnancy outcomes in women exposed to artemisinin compounds: Descriptive studies.

**Study site, year Ref**	**Antimalarial treatment**	**N (% Follow-up)**	**Outcomes of interests**		
			
			**Maternal Safety**	**Pregnancy outcomes**	
				
				**Overall**	**1^st ^trimester exposures**
China, 1976–1980 [15]	Artemisinin IM or Artemether	6 (100%)	No adverse effect	6 (100%) followed up at 5–9 year	None
TBB, 1992–2000 [16]	Artesunate alone or with mefloquine; clindamycin atovaquone-proguanil; coartemether, artemether IM	461 (89%)	No ADRs (pruritus) Maternal deaths (n = 2)*	Abortions 4.8% (n = 20) Stillbirth 1.8% (n = 7) Congenital abnormalities 0.8%: anencephaly (n = 1); midline epidermoid cyst (n = 1)	Abortions 23% (n = 10)
TBB 1999–2001 [19]	Artesunate+atovaquone-proguanil	27 (100%)	Symptoms possible ADRs: Headache (42%); muscle/joint pain (30%); abdominal pain (42%); anorexia (40%); nausea (25%); vomiting (10%); rash (15%); dizziness (70%), tinnitus (24%); contraction (15%) and sleep disturbance (8%)	Neonatal deaths (n = 1)	Normal deliveries and healthy newborns
Gambia, 1999 [41]	Artesunate + SP	325 (88%)	Maternal deaths (n = 1)**	Stillbirth (n = 11) Congenital abnormalities: umbilical hernia (n = 1); undescended testis; (n = 1) Neonatal deaths (n = 8)	Not reported
Sudan, 1997–2001 [22]	Artemether IM	28 (100%)	NR	Neonatal deaths (n = 1)	Normal delivery and healthy newborn
Sudan, 2004–2005 [23]	Artesunate + SP	32 (100%)	Giddiness and nausea (13%) Neurological exam.	Neonatal deaths (n = 1)	None
**TOTAL**	**123 1^st ^trimester, *P. falciparum *or mixed malaria 7% to 100% symptomatic 945 artemisinin exposures**	**879 (90%)**	**3 maternal deaths** **Neurological exam in 2 studies**	**Abortions n = 20** **Stillbirths (n = 18)** **Congenital abnormality: (n = 4) Neonatal deaths (n = 11)** **65 (15%) infant followed up to 1 year**	**Abortions n = 10**

* One maternal death was due to severe malaria and anaemia, the other died of causes unrelated to malaria.

** For 1 maternal death the exposure status could not be confirmed; verbal autopsies indicate that the deaths were due to postpartum haemorrhage.

Abbreviation: ADR: adverse drug reaction; AE: adverse event; IM: intramuscular injection; NR: not reported; Q: quinine; A: artemisinin derivative; A+M: artesunate+mefloquine; TBB: Thai-Burmese border

### Maternal adverse events

Overall, artemisinin derivatives were well tolerated; none of the studies reported serious adverse event (SAE) attributed to the use of artemisinin. There were seven maternal deaths reported in total: three women exposed to an artemisinin (two on the Thai-Burmese border and one during the mass drug administration campaign in the Gambia), three with unknown exposure (two reports from RCTs on the Thai-Burmese border and one in the Gambia) and one not exposed from the Gambia study. Cause of death was known for only five women [severe malaria and anaemia (1), liver abscess (1), post-partum haemorrhage (3)]. Other maternal adverse events were described clearly only in the studies conducted at the Thai-Burmese border. In these studies, adverse events that could potentially be associated with the study drug were defined as the occurrence of symptoms after treatment not present at baseline and which occurred before a recrudescence of parasitaemia or a second infection. Data from Thailand and the Thai Burmese border showed that tinnitus and dizziness were significantly less common in the artemisinin treatment group (range 9–64%) compared to the quinine arm (45–79%). Although nausea and vomiting were also significantly less frequent in the artesunate-mefloquine treatment arm in the Thailand study, no significant difference was reported in the Thai-Burmese border trials. Other adverse events were reported with a similar frequency in each treatment group. The most frequent complaints according to these studies were dizziness (range 42–45% for the artemisinin group versus 49–87% for the quinine arm); nausea (range 22–57% and 8–93% respectively); headache (range 21–30% and 30–50% respectively); anorexia (range 7–33% and 0–47% respectively) and muscle and joint pain (range 12–31% and 12–27% respectively). All of these symptoms were also reported on presentation (dizziness 57–61%; headache 72–75%; anorexia 46–62%, nausea 30%–40% and muscle/joint pain 59–68%) and so the apparent adverse events may have been due to the underlying malarial episode. Specific neurological evaluation of mothers was performed in four studies[10,12,17,22]. This included Romberg's test, assessment of heel-toe ataxia, fine finger dexterity, auditory acuity and an assessment for nystagmus. There was no report of a woman with an adverse neurological event associated with drug administration. The studies were too heterogeneous in terms of severity of disease, drug treatment and outcome measurements, and numbers to few to pool data for a meta-analysis.

### Pregnancy outcomes

Ninety-six percent of the 945 women exposed to an artemisinin in pregnancy were followed up to delivery. Twenty (2.1%) had miscarriages, 19 (2%) stillbirths and 11 (1.2%) neonatal deaths. Six (0.6%) congenital abnormalities were reported (one left aural atresia, one polythelia, one epidermoid cyst and three not described); whereas the expected rate of birth defects in developing countries is about 6% [28]. Of the 214 infants examined up to at least one year of age only one was reported to be developmentally delayed. The mother of this infant was treated with artesunate-atovaquone-proguanil and he was assessed for motor and neuro-developmental milestones (no details provided).

## Discussion

None of the studies included in this review revealed an increased risk of serious maternal adverse effects, adverse birth outcomes, or neuro-development deficits associated with the use of an artemisinin drug during pregnancy. However, these studies were not designed to assess safety endpoints and, although they were sufficiently powered to answer the original study objective, the studies were under-powered to detect rare safety outcomes, or small differences in adverse event rates, between the comparison groups.

The very low prevalence of congenital anomalies reported can partly be explained by the complex nature of birth defect ascertainment; ideally a dysmorphologist should have assessed all newborns for abnormalities that would not be detected by an untrained physician. It was also not possible to assess the expected rate of adverse birth outcomes in any of the study settings due to the lack of background population data on rates for abortions, stillbirths and congenital abnormalities. Four studies which looked for neurological damage following artemisinin exposure revealed no indication of neurotoxicity. However, in the light of recent debates about the potential ototoxicity of the artemisinins, further studies conducting thorough auditory evaluation before and after treatment may be needed [29-31].

The studies reviewed were highly heterogeneous in terms of treatment used, outcomes assessment, population and follow up rate. Different artemisinin drug regimens were used with varying control treatments as a comparator and six studies did not have a control group but compared the rates of adverse birth outcome to community rates derived from separate studies. Most of the studies used an artemisinin derivative combined with another drug, which adds to the difficulty of teasing out individual drug effects and restricts comparison between studies. Four studies used artesunate in combination with mefloquine; use of the latter in pregnancy has caused concern because of an increased risk of stillbirth in women who received this drug during pregnancy in one study [32] although this was not found in others. [33-36]. The methods used to monitor adverse event and birth outcomes also differed widely between the studies. Under-estimates of adverse outcomes cannot be ruled out, particularly for early pregnancy loss in the mass drug administration campaign in the Gambia. There are no statistics on expected rate of early pregnancy loss, and determining the prevalence of spontaneous abortion is difficult. In western countries, miscarriages occur in 10 to15% of pregnancies, mostly during the first few weeks. The prevalence of miscarriages is likely to be even higher in resource poor setting. When assessing maternal AEs, there is the methodological challenge of differentiating between the effect of malaria and its treatment which is difficult to do. Furthermore, the severity of malaria episode varied between the different study populations since three included women who had already experienced a treatment failure whilst nine others included some asymptomatic women. Furthermore, the study in the Gambia was preventive; as the majority of the participants in this study are unlikely to have had malaria, lower rates of adverse birth outcomes would be expected compared to those of studies of malaria case management. The allocation of interventions could have been influenced by many factors particularly for the non-randomized studies, such as prognostic factors (severity or malaria attack rates, parasite resistance, mother's age, gravidity, other drugs etc.), which could themselves influence birth outcome and treatment response. There is, therefore, a potential for selection and detection bias. Maternal malaria has been associated with stillbirth, abortion and LBW and these adverse end-points are also considered as possible indicators of an adverse event related to drug administration[1]. Furthermore, none of these studies controlled for other drug use, which could potentially influence pregnancy outcomes.

Most of the information on artemisinin exposure during pregnancy comes from studies conducted in Southeast Asia, at the antenatal clinics of the Shoklo Malaria Research Unit among women of the Karen ethnic minority. The RCTs had robust design and randomly allocated treatment groups, although the investigators were not blinded to the treatment group. Follow up of infants varied between these three studies (49 to 80%) and only 46 infants were followed up to one year of age and examined for developmental abnormalities in the non-comparative studies. The women enrolled in these studies are likely to be representative of this population group (Karen ethnic minority) as over 90% of the women in the studied Thai-Burmese border region attend ANCs. These findings cannot be extrapolated directly to sub-Saharan Africa considering geographic differences in parasite species, resistance pattern, transmission intensity and host immunity.

A number of studies of artemisinin treatment in pregnancy are in progress (Table 4), which will contribute further to knowledge on the safety of these drugs. However, except for the phase IV study being conducted by the Centres for Disease Control in Tanzania, these studies focus on 2^nd ^and 3^rd ^trimester pregnancies. The results of animal studies suggest that if there is a safety issue related to the administration of artemisinins in pregnancy this is likely to occur very early in pregnancy. Only 123 documented first trimester exposures have been reported and this does not provide enough evidence to determine safety. Moreover, an "all or nothing rule" seems to apply for exposure during the first weeks of pregnancy. Animal experiments suggest that congenital abnormalities occur only after exposure over a narrow dose range and over a limited period of time and that exposure usually lead to a normal pregnancy outcome or death of the foetus. Developmental toxicity studies in the monkey confirmed findings from rodent studies; with embryo death induced at therapeutic dose ranges [37]. The teratogenic effect is thought to involve red blood cells production, erythropoiesis, which implies the human sensitive period would be within the first trimester of pregnancy [38]. An effect of ACTs on early pregnancy loss will be difficult to detect, especially in communities where artemisinins are likely to be used most frequently. More extensive studies are needed that will be able to detect rarer outcomes and any congenital abnormalities that might result from exposure in the first trimester of pregnancy. Ethical constraints will prohibit randomized trials of artemisinins in the first trimester of pregnancy in most communities where alternatives exist until more information on safety has been obtained. Thus, data will largely have to come from observational studies.

**Table 4:** Ongoing Studies (Information extracted from Malaria in Pregnancy (MiP)* Database [42] on 05/02/2007).

**Study Setting**	**Design & Drug Regimen**	**Outcome**	**Status** (start date-completion date)
Safety/Efficacy prevention trial in pregnancy			

Ifakara, Tanzania CDC/IHRDC-IMPACT	Randomised open label, n = 1200 (400 per arm) IPTp Control: SP 2 doses Intervention: 1. SP monthly 2. SP+artesunate monthly	1°: Placental parasitaemia and AEs 2°: Maternal illness and parasitaemia at delivery, birth outcome (BW, Gestational age, foetal and infant health), childhood developmental milestones	Recruitment concluded; ongoing follow up (01/03-ongoing)

Safety/Efficacy Treatment trial in Pregnancy			

ANC at Muheza Hospital, Tanzania GMP	Randomised open label, target (Phase III) n = 350 2^nd ^or 3^rd ^trimesters Control: SP Intervention: SP+amodiaquine, Amodiaquine+artesunate, Chloroporguanil-dapsone	1°: Treatment failure at day 28; Treatment outcome (parasite/fever clearance, parasite recrudescence) 2°: Foetal viability and birth outcomes (preterm delivery, foetal death, perinatal/neonatal mortality, neonatal abnormality); maternal AE (hypoglycaemia)	Recruitment completed (01/04–07/06)
Shoklo Malaria Research Unit (SMRU) ANC, Thailand UNICEF-UNDP-World Bank-WHO-TDR	Randomized intervention trial n = 250 2^nd ^or 3^rd ^trimesters Group 1: Artesunate Group 2: Co-artemether (artemether/lumefantrine)	1°: Treatment outcome at day 42 or at delivery (parasite/fever clearance, parasite recrudescence) 2°:Gametocyte carriage; pharmacokinetic parameters; histo-pathology examination of the placenta	Currently recruiting (06/02/2004–31/12/2008)
Bangladesh WHO	Randomised controlled trial n = 684 Control: placebo rectal capsule Intervention: Artesunate rectal capsule	Pregnancy outcomes	Currently recruiting (10/11/2003-ongoing)
Malawi; Prof Meshnick, UNC	Randomised open label, n = 141 2^nd ^or 3^rd ^trimesters Control: SP Intervention: SP+artesunate or SP+azithromycin	1°: Parasitological failure rates; parasite clearance time; fever clearance times and incidence rate of adverse events 2°: Prevalence rate of abortions; still births; peripheral parasitaemia at delivery; placental malaria and of maternal anaemia	Recruitment completed (09/2003–10/2005)

Efficacy/Pharmacokinetic trial in Pregnancy			

Mozambique UCT, South Africa	Non-Randomized openLabel, target n = 30 2^nd ^or 3^rd ^trimester pregnant HistoricalControl Intervention: SP+artesunate	1°: Pharmacokinetic parameters 2°: gametocyte carriage, maternal AE & birth outcomes	Currently recruiting (03/2006–09/2008)
Kinshasa, DRC, NIH-NICHD	Dose-equivalence trial: part of investigational new drug application n = 60 2^nd ^or 3^rd ^trimester Control: SP Intervention: Artesunate-mefloquine combinations	Pharmacokinetic parameters	Recruitment completed (07/2005–12/2005)

Pharmacovigilance: Post-marketing surveillance			

Tanzania, CDC	Pharmacovigilance surveillance system: part of a large ongoing study to look at district wide use of ACTs 1^st ^trimester Control: SP Intervention: SP+Artesunate	Pregnancy outcome and status of child	Ongoing (2005–2007)
A partnership between Novartis WHO-TDR and the Government of Zambia. [43]	Pregnancy Registry Prospective active surveillance cohort. Expected n = 1600 Control: SP Intervention: artemether-lumefantrine	Maternal and neonatal outcomes examined	Ongoing (2005)

* MiP is a consortium of experts in the field of malaria funded by the Bill and Melinda Gates Foundation to review current research and develop future research strategy for malaria in pregnancy. One of the key objectives of MiP is to create a database containing all published and unpublished research and a trial registry on malaria in pregnancy.

Abbreviations: ACT: artemisinin combination therapy; AE: adverse events; ANC: antenatal care; BW: birthweight; CDC: Centers for Disease Control & Prevention; GMP: Gates Malaria Partenership; DRC: Democratic Republic of the Congo; IHRDC: Ifakara Health Research and Development Centre; IPTp: intermittent presumptive treatment for pregnancy; NIH-NICHD: National Institutes of Health-The National Institute of Child Health and Human Development; RCT: randomized controlled trials; SP: sulphadoxine-pyrmethamine; TBB: Thai-Burmese Border; UNC: University of North Carolina; UCT: University of Cape Town; WHO-TDR: World Health Organization – The Special Programme for Research and Training in Tropical Diseases

Unfortunately, most countries in sub-Saharan Africa do not have the infrastructure and resources for routine pharmacovigilance and very few have a formal system for routine collection of data on possible drug related adverse effects [39]. In industrialized countries, a variety of post-marketing surveillance techniques is used. Case reports and case series are the first source of information to detect adverse events in pregnancy. Although these are useful in the generation of hypotheses of a possible safety signal, specific pharmaco-epidemiological studies using a cohort or case-control approach are required to evaluate teratogenic risk. The main factors impeding the implementation of pharmacovigilance in poor countries include limited access to healthcare facilities, availability of most prescription drugs from the informal market, poor labelling of medicines, counterfeit and sub-standard pharmaceutical products, a high level of illiteracy, poor record keeping and a shortage of qualified healthcare professionals. Special pharmaco-epidemiological studies will be needed to assess the safety profile of a product's use outside the controlled environment of clinical trials. Active surveillance systems could use the existing sentinel demographic surveillance sites (DSS), which already undertake regular household visits to obtain more information on drug usage and adverse effects during pregnancy. However, these studies are expensive and not sustainable in the long term and should be restricted to newer products or following identification of new safety concerns for an older drug. Antenatal clinics (ANC) could make a useful platform for routine surveillance, as a high proportion of pregnant women attend an ANC at least once during pregnancy in sub-Saharan Africa [40]. It is necessary to develop a more pragmatic pharmacovigilance system that can be linked to a routine health information system and ideally a mix of different approaches should be used to assess the safety profile of individual drugs for pregnant patients.

## Conclusion

Malaria in pregnancy is a major public health issue and infected pregnant women need prompt treatment with effective drugs. Although artemisinin derivatives and combinations have an excellent efficacy profile, there is very limited data on the safety of artemisinin use during pregnancy particularly in sub-Saharan Africa. Although a few studies of the safety and efficacy of artemisinins during pregnancy are currently underway, these will not produce data on the safety of artemisinins during the first trimester of pregnancy. Post-marketing pharmacovigilance of artemisinin use during pregnancy is needed urgently as ACT is implemented in almost all countries in Africa. Innovative pharmacovigilance tools, methods and systems are needed to monitor the safety of artemisinins and other antimalarials.

## Authors' contributions

SD carried out the literature review and wrote the first draft of the paper. SH, DC and BG contributed to the structure and content and were involved in re-drafting the paper.
